# Hydrogel-extraction technique for non-invasive detection of blue fluorescent substances in plant leaves

**DOI:** 10.1038/s41598-022-17785-w

**Published:** 2022-08-10

**Authors:** Shigeyuki Iwasa, Yuso Kobara, Katsumi Maeda, Kuniaki Nagamine

**Affiliations:** 1 Research Center for Organic Electronics, Yamagata University, Yamagata, 992-8510 Japan; 2 System Platform Research Laboratories, NEC Corporation, Chiba, 270-1198 Japan; 3 Institute for Agro-Environmental Sciences, National Agriculture and Food Research Organization, Ibaraki, 305-8604 Japan; 4 Graduate School of Organic Materials Science, Yamagata University, Yamagata, 992-8510 Japan

**Keywords:** Plant physiology, Plant signalling, Bioanalytical chemistry, Sensors

## Abstract

This paper reports a new hydrogel extraction technique for detecting blue fluorescent substances in plant leaves. These blue fluorescent substances were extracted by placing a hydrogel film on the leaf of a cherry tomato plant infected with *Ralstonia solanacearum*; herein, chlorogenic acid was confirmed to be a blue fluorescent substance. The wavelength at the maximum fluorescence intensity of the film after the hydrogel extraction was similar to that of the methanolic extract obtained from the infected cherry tomato leaves. Chlorophyll was not extracted from the hydrogel film because no fluorescence peak was observed at 680 nm. Accordingly, the blue fluorescence of the substances extracted from the hydrogel film was not quenched by the strong absorption of chlorophyll in the blue light region. This hydrogel extraction technique can potentially detect small amounts of blue fluorescent substances and the changes in its amount within the leaves of infected plants. These changes in the amount of blue fluorescent substances in the early stages of infection can be used to detect presymptomatic infections. Therefore, hydrogel extraction is a promising technique for the noninvasive detection of infections before onset.

## Introduction

Plant diseases induced by pathogenic infections cause major losses in the agricultural industry worldwide. Generally, the suppression of diseases with pesticides and fungicides is difficult after the onset of infection. Moreover, pathogenic infections often spread rapidly over large areas of the field. Early detection of these infections before onset with appropriate management strategies, such as pesticide application and removal of small infected areas, can enable disease control and improve agricultural productivity.

Currently, plant infections are detected by enzyme-linked immunosorbent assays (ELISA) or polymerase chain reactions (PCR)^1,2^. However, these detection techniques are applied after the onset of a plant disease and require a preprocess involving plant destruction, such as grinding the leaves in a mortar. In addition, they are usually expensive and time-consuming. Meanwhile, research on image-based detection using machine learning has been actively conducted for the past decade aiming for automated plant diagnosis^3,4^. However, most of the research results were limited to the laboratory environment due to the difficulty in obtaining training data applicable to the field^4^.

Pathogenic infections produce various stress responses in plants^5^. When a pathogenic infection is recognized, the amount of some antibacterial substances, which are originally present in small amounts in plants, increases. These substances are called phytoanticipins^6^. Antioxidants such as caffeic acid and chlorogenic acid, which are also phenylpropanoids, are well-known phytoanticipins^7–11^. Additionally, in infected plants, the accumulation of salicylic acid, a phytohormone, induces systemic acquired resistance as an immune function^12–15^. During pathogenic infections, plants are known to accumulate specific phenolic compounds, such as phenylpropanoids^7–11^ and salicylic acid^12–15^.

Under UV excitation, plants emit a wide fluorescence spectrum, ranging from approximately 400 to 800 nm. This spectrum comprises two distinct types of fluorescence: a blue-green fluorescence (BGF) characterized by a peak at approximately 440 nm (F440) with a shoulder near 520 nm (F520) and a fluorescence in the red and far-red regions with characteristic peaks at approximately 680 nm (F680) and 740 nm (F740), respectively^16–21^. BGF in intact leaves is emitted by cinnamic acids, mainly ferulic acid^22,23^ covalently bound to the cell walls of the epidermis. In addition, BGF can also emanate from other secondary metabolites including phenylpropanoids, such as *p*-coumaric acid^23^, caffeic acid^23^, scopoletin^24–26^, and chlorogenic acid^27,28^. Therefore, BGF intensity can be increased by increasing the amount of phenylpropanoid compounds in plants^24–30^.

Non-invasive techniques for imaging the patterns of multispectral fluorescence in infected leaves have been studied; these techniques can reportedly detect stress and disease events^24–26,28–31^. Changes in UV-induced blue (F440) and green fluorescence (F520) in pathogen-challenged plants were measured by multicolor fluorescence imaging. These changes were related to the up-regulation of plant secondary metabolism and an increase in phenolic compounds involved in plant defense^26,28–31^. However, in green mesophyll cells, blue light is strongly absorbed by chlorophylls^17–20^, which indicates that blue fluorescence intensity is largely affected by fluorescence quenching caused by chlorophylls.

Non-invasive extraction techniques using hydrogels have been studied for detecting sweat components in the human body^32–34^. This sensor device was composed of an electrochemical biosensor (e.g., l-lactate sensor and Cl^−^ ion sensor) covered with an agarose gel film. When the gel film came in contact with human skin, the sweat was continuously extracted into the gel and then electrochemically measured using the biosensor. Biocompatible and mechanically stable agarose (Fig. S1) is one of the suitable hydrogels for the extraction of chemical compounds in the living body because it has a relatively large size of pore structure with a diameter ranging from 0.1 to 1  µm^35^. Similar hydrogel touchpad-based biosensors have been evoked over the past few years and showed their applicability to the other sweat components (glucose^36,37^, cortisol^38^, l-dopa^39^, and pH^40^). These hydrogel-based extraction techniques are quite important to non-invasively analyze the chemical-based physiological information in the living body.

The surface of leaves is commonly covered with a cuticle membrane, which is 0.1–10 μm or more in thickness^41^. The outermost layer of the cuticle membrane is composed of cuticular wax, which is a thin hydrophobic layer consisting of aliphatic hydrocarbons, such as alkanes, alcohols, aldehydes, and fatty acids, with typical carbon chain lengths of 20–40 carbon atoms (C20–C40)^41–45^. The cell wall underneath the cuticle is filled with an interstitial fluid called apoplast^46^. The main function of the cuticle is to protect the leaf against water loss and various environmental stresses^41–45^. However, substances such as carbohydrates, amino acids, hormones, or phenols inside plants can be released externally through defects such as micropores and micro-damaged areas, trichome bases, leaf tips, and edges^42^. Moreover, aqueous solutions can be transported through stomata as a result of aerosols deposited on the leaf surface, which changes the hydrophobicity of the leaf surface^38^. These releases of substances from the inside of plants are known as leaching phenomenon.

**Figure 1:**
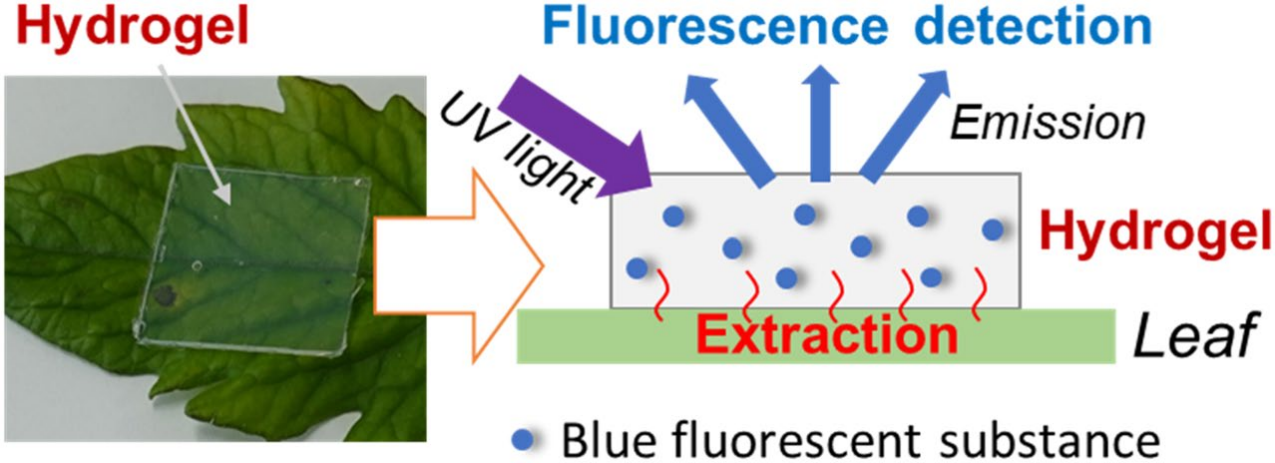
Conceptual illustration of the hydrogel extraction and fluorescence detection of blue fluorescent substances in plant leaves.

This paper reports a new hydrogel extraction technique for detecting blue fluorescent substances in plant leaves. Agarose gel was used as a hydrogel since the gel made from plant-derived agar is a plant- and environmentally friendly and inexpensive. This hydrogel extraction technique involves the non-invasive extraction of plant components using a hydrogel film. Water-soluble components inside plants are selectively extracted into the hydrogel film on leaves based on the leaching phenomenon, and the phenolic compounds with blue fluorescence in the film are detected by UV light irradiation (Fig. 1). The blue fluorescence used in this technique is unaffected by fluorescence quenching of chlorophylls, which is an inhibitory factor for the quantification of blue fluorescence. However, this hydrogel film does not extract chlorophylls. This study demonstrated an extraction and fluorescence detection technique using a hydrogel film for monitoring salicylic acid in tomato leaves after absorbing salicylic acid through roots. Water-soluble phenolic substances in the leaves of bacteria-infected cherry tomato plants were also analyzed using this technique.

## Results and discussion

### Hydrogel extraction of salicylic acid in leaves

**Figure 2:**
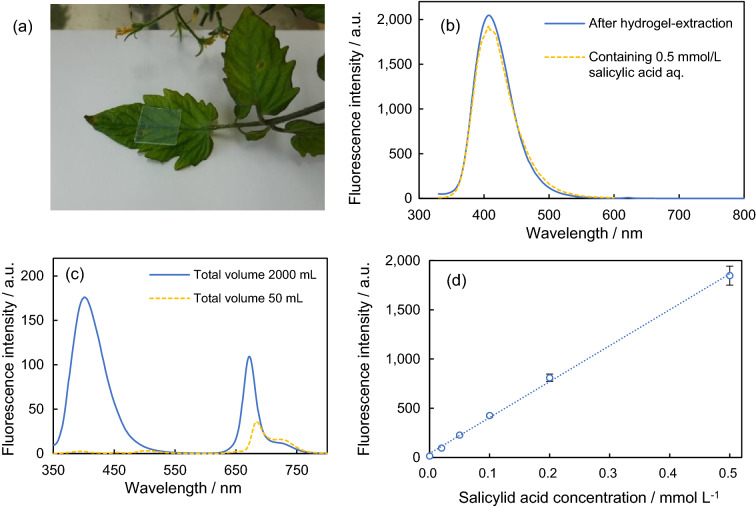
(**a**) Photograph of hydrogel extraction: a hydrogel film extracting salicylic acid from a cherry tomato leaf. (**b**) Fluorescence spectra of a hydrogel film after its placement on a cherry tomato leaf for 3 h (blue line); the roots of this plant were immersed in an aqueous solution of salicylic acid (7 mmol/L) for 24 h. The yellow-dashed lines indicates the spectra of a hydrogel film containing 0.5 mmol/L of salicylic acid aqueous solution. (**c**) Fluorescence spectra of the methanol extract of a cherry tomato leaf (0.5 g). The roots of this cherry tomato plant were immersed in 7 mmol/L salicylic acid aqueous solution for 24 h. Total solution volume: 2000 mL (blue line), 50 mL (yellow dashed line). (**d**) Relationship between salicylic acid concentration and fluorescence intensity (410 nm) for a hydrogel film containing an aqueous solution of salicylic acid. Fluorescence emissions were obtained using UV light (Xenon lamp, 310 nm).

After the roots of cherry tomato plants were immersed in a 7 mmol/L salicylic acid aqueous solution for 24 h, the hydrogel films were placed on their leaves for 3 h (Fig. 2a). In the beginning, the hydrogel films were lightly pressed on the leaf with a finger because the films were slippery on the leaf surface with hydrophobicity. Figure 2b shows the fluorescence spectrum of a hydrogel film after its placement on a leaf for 3 h. The fluorescence spectrum was almost the same as that of a hydrogel film containing 0.5 mmol/L of salicylic acid. Salicylic acid was assumed to diffuse into the hydrogel film from the inside of the leaves through defects such as micropores and micro-damaged areas, or trichome bases. Chlorophyll was not extracted from the hydrogel film because no fluorescence peak was observed at 680 nm. This indicates that the fluorescence quenching of chlorophyll had no effect on the blue fluorescence of the substances extracted from the hydrogel. The extraction of salicylic acid through hydrogel films was carried out on tobacco leaves, which are thicker than tomato leaves (Fig. S2a). Salicylic acid was absorbed through the roots from soil containing an aqueous solution of salicylic acid (7 mmol/L) for 24 h. The fluorescence spectrum of the hydrogel film was similar to that of a hydrogel film containing a 0.2 mmol/L aqueous solution of salicylic acid (Fig. S2b). The cherry tomato and tobacco leaves showed different concentrations of salicylic acid in the hydrogel films. For tobacco, the salicylic acid concentration in the soil was diluted because the soil contained water. In addition, the type and age of the plant may affect the concentration of substances absorbed through the roots. The hydrogel film extracted salicylic acid from the inside of the leaf and likely extracted other water-soluble substances in the leaf, excluding the cell walls.

Figure 2c shows the fluorescence spectrum of the methanolic extract obtained by grinding the cherry tomato leaves (0.5 g) in a mortar. This plant was allowed to absorb salicylic acid through the roots. The intensity of the blue fluorescence changed significantly depending on the amount of diluted extract. In this spectrum, the fluorescence around 400 nm almost disappeared for 50 mL of the methanolic extract; this observation indicates the strong influence of fluorescence quenching by chlorophyll on the fluorescence in the blue light range. To quantitatively analyze the amount of salicylic acid in the leaf, the extract was diluted with a large amount of methanol (2000 mL). Moreover, the dilution reduced the fluorescence quenching of chlorophyll. The amount of salicylic acid in the leaf was found to be 3.9 mg/g-leaf weight or 0.033 mg/cm^2^-leaf area. This amount was calculated using average leaf weight per area and the salicylic acid content in the extract (Table 1) obtained from the calibration curve of fluorescence intensity at 410 nm depending on salicylic acid concentration (Fig. S3).

**Table 1:** Salicylic acid content per leaf area and the factors used for calculation.

Salicylic acid content extracted from a leaf 0.5 g (mmol)		0.014
Salicylic acid content per leaf weight (g/g-leaf)		0.0039
Average leaf weight per area (g/cm^2^)		0.0083
Salicylic acid content per leaf area (g/cm^2^)		3.3 × 10^–5^

For the hydrogel film, the relationship between salicylic acid concentration and fluorescence intensity (Fig. 2d) showed good linearity. The linear correlation coefficient was 0.999. The standard deviations (SDs) (*n* = *5*) were 3–6% for the concentration of 0.02–0.5 and 10% for the blank sample. The limit of detection (LOD) of salicylic acid determined from the three SD/slope was 1.0 μmol/L, which indicates that the minimum amount of salicylic acid detectable was 13.8 ng/cm^2^-hydrogel film. The amount of salicylic acid extracted from cherry tomato leaves through hydrogel films was calculated to be 0.0072 mg/cm^2^ using the salicylic acid concentration in a hydrogel film obtained from the calibration curve (Fig. 2d) and the volume of hydrogel film (Table 2). Consequently, the extraction rate of salicylic acid from the leaf to the hydrogel film was estimated to be 23%. We also confirmed that the water immersion of a leaf in a plastic tube (Fig. S4) leads to the extraction of salicylic acid from the leaf with high efficiency (approximately 14%). From the viewpoint of the operation in the field, the hydrogel extraction is more practical than the water immersion technique requiring the water immersion and the fluorescence measurement with the quartz cell, since the hydrogel extraction is a simple detection method consisting of placing a hydrogel film on a leaf and the UV light irradiation to the leaf surface (Fig. 1).

**Table 2:** Salicylic acid content in a hydrogel film and the factors used for calculation.

Volume of hydrogel film (cm^3^)		0.4
Water volume ratio in hydrogel film (–)		0.96
Salicylic acid concentration in a hydrogel film (mmol/L)		0.55
Salicylic acid content in a hydrogel film (g/cm^2^)		7.2 × 10^–6^

### Blue fluorescent substances in the leaves of infected cherry tomato plants

**Figure 3:**
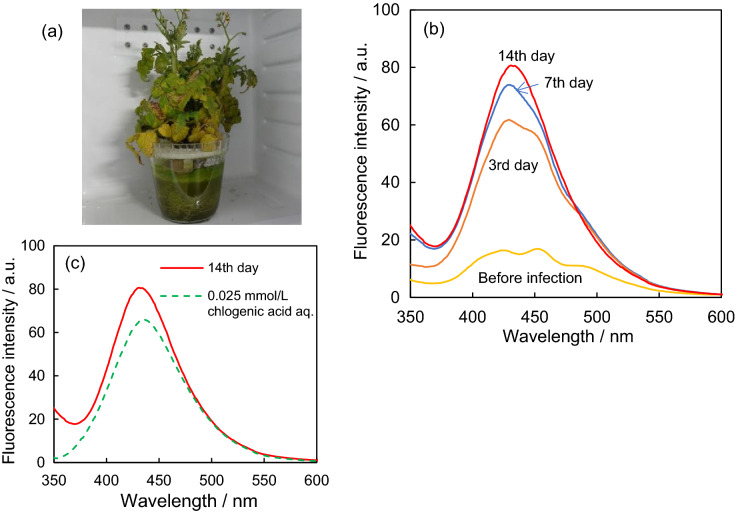
(**a**) Photograph of a cherry tomato hydroponically grown on an aqueous solution containing *Ralstonia solanacearum* at 35 °C for 7 days. (**b**) Fluorescence spectra for the methanol extract of a cherry tomato leaf before and after the 3rd, 7th, and 14th day. This plant was grown using an aqueous solution containing *Ralstonia solanacearum* at 35 °C. (**c**) Fluorescence spectra for the 0.025 mmol/L chlorogenic acid methanol solution (green-dashed line) and the methanolic extract of a cherry tomato leaf on the 14th day after being grown on an aqueous solution containing *Ralstonia solanacearum* at 35 °C (red line). Fluorescence emissions were obtained using UV light (Xenon lamp, 310 nm).

Figure 3a shows a cherry tomato plant grown hydroponically in an aqueous solution containing *Ralstonia solanacearum* at 35 °C. In the fluorescence spectra of the methanolic extract of leaves of cherry tomatoes (Fig. 3b), the intensity between 400 and 500 nm was higher than that obtained before and after being grown on an aqueous solution containing *Ralstonia solanacearum,* and increased during the infection period between 3rd and 14th days. There was no significant change in the fluorescence intensity of chlorophyll (680 nm), which affects blue fluorescence intensity, in the early stages of infection (up to the 3rd day). The amount of blue fluorescent substances in the leaves is considered to increase after the infection with *Ralstonia solanacearum*. An increase in intensity does not necessarily reflect the quantitative increase in the amount of blue fluorescent substances because the amount of chlorophyll may change during the infection period between 3rd and 14th days. Furthermore, the composition of the blue fluorescent substances was probably not constant because of changes in the spectral shape and peak top wavelength.

**Figure 4:**
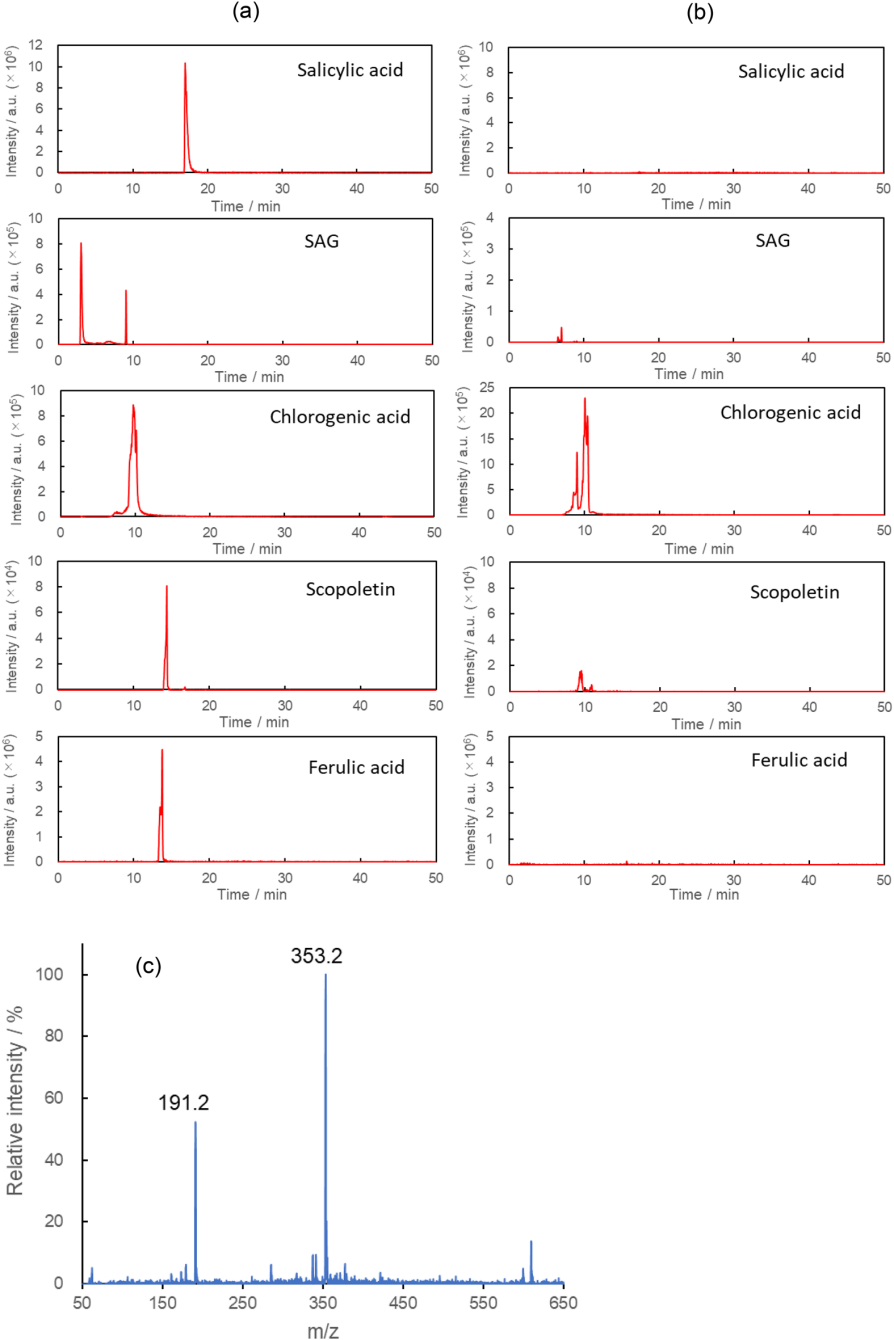
(**a**) Extracted ion chromatograms (EICs) of a mixed standard solutions containing candidate compounds including salicylic acid, salicylic acid 2-*O*-β-d-glucoside (SAG), chlorogenic acid, scopoletin, and ferulic acid at a concentration of 5 ppm. (**b**) Extracted ion chromatograms (EICs) and (**c**) MS spectra of the eluted components at 10 min in LC for the methanolic extract of cherry tomato leaves on the 14th day after being grown on an aqueous solution containing *Ralstonia solanacearum* at 35 °C.

The methanolic extracts of the leaves were analyzed using liquid chromatography-tandem mass spectrometry (LC–MS/MS) on the 14th day of infection for the identification of components. In previous studies, salicylic acid^12–15^, salicylic acid 2-*O*-β-d-glucoside (SAG)^47^, chlorogenic acid^27–29^, scopoletin^24–26^, and ferulic acid^22,23^ were regarded as candidate compounds with increased blue fluorescence after bacterial infections. The chemical structures of these compounds are shown in Fig. S5. Figure 4a,b show the extracted ion chromatograms (EICs) of mixed standard solutions containing these compounds and the methanolic leaf extracts, respectively. In the leaf extract spectra, a pseudomolecular ion [M-1]^−1^ corresponding to chlorogenic acid (MW 354) was observed. The retention time of 10 min was consistent with that of chlorogenic acid in a mixed standard solution. The pseudomolecular ions [M-1]^−1^ for chlorogenic acid and the fragment (MW191, Fig. S6) were observed in the MS spectrum during elution at a retention time of 10 min in LC (Fig. 4c). The concentration of chlorogenic acid in the methanolic extract was found to be approximately 10 ppm compared with a mixed standard solution containing 5 ppm chlorogenic acid. The amount of chlorogenic acid in the infected cherry tomato leaves was calculated as 0.24 mg/g-fresh leaf after an infection period of 14 days. In a previous study^48^, the amount of chlorogenic acid in the leaves of a tomato plant infected with *Clavibacter michiganense* was reported to be 200–500 nmol/g-fresh leaf (0.07–0.18 mg/g-fresh leaf) after 5 weeks of infection. The amount of chlorogenic acid (0.24 mg/g) found in this study is similar to that of the previous study. The wavelength of the maximum fluorescence intensity of the leaf on the 14th day of infection was almost the same as that of 0.025 mmol/L chlorogenic acid. Chlorogenic acid is one of the blue fluorescent substances in the leaf after infection. Although a component of [M-1]^−1^ corresponding to scopoletin was observed, it was assumed to be a fragment of chlorogenic acid (Fig. S6). This is because its retention time agreed with that of chlorogenic acid in a mixed standard solution. Among the other candidate compounds, salicylic acid and ferulic acid were not detected in the EICs of the leaf methanolic extract. Although a component of [M-1]^−1^ corresponded to SAG in the EIC of the leaf extract, it was not identified as SAG and did not contribute to blue fluorescence of the leaf extract due to low intensity. On the 14th day, the wavelength corresponding to the maximum intensity in the leaf spectrum almost coincided with that in the spectrum for 0.025 mol/L chlorogenic acid in a methanol solution (Fig. 3c). However, the wavelength at the maximum intensity did not match exactly in either spectrum. This result implies that, in addition to chlorogenic acid, other substances also contributed to the blue fluorescence. However, these substances could not be identified in this study.

### Hydrogel extraction from leaf of infected cherry tomatoes

**Figure 5:**
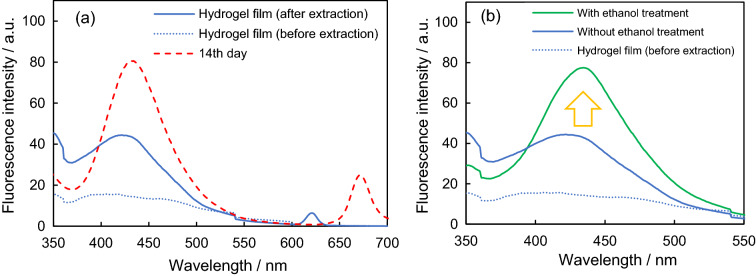
(**a**) Fluorescence spectra of a hydrogel film before (blue dot line) and after (blue line) its placement on an infected cherry tomato leaf for 3 h. This cherry tomato plant was grown using an aqueous solution containing *Ralstonia solanacearum* at 35 °C for 14 days. Fluorescence spectra of the methanolic extract (red dashed line) of a cherry tomato leaf. (**b**) Fluorescence spectra of hydrogel films after the hydrogel-extraction with ethanol treatment (green line) and without ethanol treatment (blue line). Ethanol treatment: a drop of ethanol was applied to the surface of leaves using a glass pipet before the extraction. The spectrum of a hydrogel film before the extraction is also shown in the background (blue dot line). Fluorescence emissions were obtained using UV light (Xenon lamp, 310 nm).

Hydrogel film extraction of a cherry tomato leaf was performed for 3 h. These plants were grown using an aqueous solution containing *Ralstonia solanacearum* at 35 °C for 14 days. After hydrogel extraction, the film showed blue fluorescence with a maximum intensity at 428 nm (Fig. 5a), indicating that the hydrogel film extracted the blue fluorescent substances from the inside of the leaf. Blue fluorescent substances were considered to diffuse through defects of the cuticle into the aqueous medium of the hydrogel (Fig. S7). On the other hand, chlorophylls inside plant cells did not diffuse into the hydrogel due to the insolubility in water. The wavelength corresponding to the maximum intensity obtained for the hydrogel film and that of the leaf extract differed by approximately 5 nm. The reason for this slight difference in wavelengths at maximum intensity was that the composition of the extracted substances did not exactly match owing to the different extraction solvents used. Weak fluorescence at 620 nm was secondary to excitation (310 nm) owing to the double wavelength.

Before the hydrogel extraction, a drop of ethanol was applied on the surface of the leaves using a glass pipet and wiped off lightly with paper. This ethanol treatment improved the adhesion between the hydrogel film and leaf surface by decreasing the cuticular wax, which is a hydrophobic layer. After the ethanol treatment, the hydrogel film was found to be less slippery than before. Consequently, the blue fluorescence observed was approximately twice that before the ethanol treatment (Fig. 5b). This result suggests that the extraction efficiency of the hydrogel film can be increased by improving its adhesion on the hydrophobic leaf surface. Figure S8 shows fluorescent spectra of hydrogel films after the extraction of the middle part of leave of different branches, and the tip and middle parts of a leaf. The presence of blue fluorescent substances in different leaves and different parts of the leaves of the infected cherry tomato was confirmed by the hydrogel extraction. For the discussion on the quantity and quantitative change of blue fluorescent substance in leaves, the optimization of the hydrogel such as adhesion to the leaf is required for dealing with the extraction variability.

In our study, hydrogel extraction was performed on cherry tomato leaves on the 14th day after infection. A previous study reported that chlorogenic acid levels in tobacco leaves increased within 15 h of inoculation with *Pseudomonas syringae pathovars*^10^. Detection techniques for very small amounts of blue fluorescent material are most effective in the early stages of infection and have the potential to detect presymptomatic infections for many crops as well as tomatoes. Hydrogel extraction is a promising non-invasive technique for the detection of infections before onset. However, issues such as the improvement of adhesion between the hydrogel film and the hydrophobic leaf surface remain.

## Methods

### Plant material

The cherry tomatoes “Regina”, *Solanum lycopersicum* cv. Regina (Sakata Seed), were grown indoors in water culture pots (Yamato Plastic) near a south-facing window with an aqueous solution containing commercial fertilizer (Fine Powder Hyponex, Hyponex, Japan). The cherry tomatoes “Yellow-mimi”, *Solanum lycopersicum* cv. Yellow-mini, were grown outdoors in pots containing commercial potting soil (Tachikawa Heiwa Nouen). A branch with some leaves, which was approximately 50 cm long, was cut off from a 4-month-old cherry tomato “Yellow-mimi”, plant. Subsequently, the lower part of the branch was immersed in water for 7 days in order to newly root. Tobacco, *Nicotiana tabacum L.* cv. Tsukuba ichi-gou (Japan tobacco), was grown indoors under LED illumination (approximately 3000 lx) for 12 h per day in pots containing commercial potting soil.

### Absorption of salicylic acid into plants

Salicylic acid was absorbed by plants through the roots. Herein, salicylic acid was used as a model water-soluble substance inside the leaves. (1) The roots of a rooted branch of a cherry tomato “Yellow-mimi” plant were immersed in an aqueous solution of salicylic acid (7 mmol/L) for 24 h. (2) Three hundred mL of 7 mmol/L salicylic acid solution was poured over the soil of the 2-month-old tobacco plant 24 h before the hydrogel-extraction. Hydrogel extraction was performed on cherry tomatoes and tobacco by placing hydrogel films on the leaves for 3 h. Water-immersion extraction was performed on a cherry tomato by immersing approximately half of a leaf in 1.5 mL of water in a polypropylene tube for 3 h (Fig. S4).

### Hydrogel film

A 1 mm-thick framework of polytetrafluoroethylene sheets (Nichias), which had a 2 cm × 2 cm square cutout, was placed on a slide glass. A mixture of agar powder (0.8 g, gelling temperature 30–31 °C, Nakalai Tesque) and pure water (19.2 mL) was heated in the microwave oven to prepare a 4 w/v% agar aqueous solution. The heated agar aqueous solution was dropped into the cutout space of the framework on a glass slide. The framework with the agar aqueous solution was sandwiched between the glass slides. Two glass slides sandwiching the framework were fixed with two clips and kept for an hour at room temperature. The agar aqueous solution resulted in a hydrogel film with the dimensions of 2 × 2 cm square and 1 mm thickness.

### Extraction of leaf component

The cherry tomato leaf sample (0.5 g) was ground with 10 mL methanol (Fujifilm Wako Pure Chemical, Tokyo, Japan) in a mortar. The methanol-containing ground leaves were placed in a polypropylene tube and centrifuged at 10,000×*g* for 5 min. The supernatant was separated from the tubes. The remaining precipitate in the centrifuge tube was mixed with methanol and shaken. This mixture was centrifuged again at 10,000×*g* for 5 min. After all supernatants were combined and diluted with methanol to the required amount, the methanol solution was used to measure the fluorescence spectra.

### Bacterial infection with *Ralstonia solanacearum*

*Ralstonia solanacearum* used in this study was obtained from a field of cherry tomatoes (Shizuoka, Japan) and grown in a selective broth medium. Bacterial broth of *Ralstonia solanacearum* at a cell number of over 6.7 × 10^8^ cfu/mL was diluted 10 times with tap water. Commercial fertilizer (Fine Powder Hyponex, Hyponex, Japan) was added to the bacterial aqueous solution. The bacterial concentration in the aqueous solution was confirmed to be over 10^6^ cfu/mL using a commercial immunochromatography kit for *Ralstonia solanacearum* (ImmunoStrip^®^, Agdia). Three-month-old cherry tomato “Regina” was hydroponically grown in the bacterial aqueous solution under LED illumination (approximately 3000 lx for 12 h per day) at 35 °C in a thermostatic chamber for 14 days. The number of days in which the roots were immersed in the bacterial solution was regarded as the infection period. On the 3rd, 7th, and 14th day of infection, a leaf sample of 0.5 g was extracted with methanol, and the extract was diluted to a total of 100 mL of methanol solution. This methanol solution was used for recording the fluorescence spectra. On the 14th day, the leaf components were extracted using hydrogel films for 3 h.

### Characterization

Fluorescence spectra of the leaf extract or hydrogel film were measured using an FP-8600 spectrofluorometer (Jasco) at an excitation wavelength of 310 nm. The slit width was set to 10 and 5 nm for the excitation and emission monochromators, respectively. A film holder (FHL-809, Jasco) was used to measure the fluorescence spectra of the hydrogel films.

The components of the leaf extract obtained from a bacteria-infected cherry tomato plant “Regina”, were analyzed using liquid chromatography-tandem mass spectrometry (LC–MS/MS) (XEVO-TQD, Waters) operating in negative ion electrospray mode. Chromatographic separation of analytes was carried out on a reversed-phase separation column (Discovery HS F5, particle size: 3 μm, length × inner diameter: 150 × 2.1 mm, Supelco) using a mixture solvent of acetonitrile/water containing 0.1% formic acid as the mobile phase. Here, the acetonitrile concentration was graded from 0% at the beginning to 100% at the end over 60 min at a flow rate of 1.0 mL/min at 40 °C. Extracted ion chromatograms (EICs) obtained by LC–MS/MS were used to identify the leaf components. Salicylic acid (Fujifilm Wako Pure Chemical), salicylic acid 2-*O*-β-d-glucoside (SAG) (Toronto Research Chemicals Inc.), chlorogenic acid (MP Biomedicals, Inc.), scopoletin (Fujifilm Wako Pure Chemical), and ferulic acid (MP Biomedicals, Inc.) were selected as candidate compounds with blue fluorescence in the methanolic extract. A mixed standard solution containing the candidate compounds at a concentration of 5 ppm was used for identification.

## Supplementary Information

Supplementary Information.

## Data Availability

All data generated or analyzed during this study are included in this published article and its Supplementary Information Files.
