# Supplementary material for: Hydrogel-extraction technique for non-invasive detection of blue fluorescent substances in plant leaves
Source: Sci Rep. 2022 Aug 10;12:13598. doi: 10.1038/s41598-022-17785-w (PMC9365774; doi:10.1038/s41598-022-17785-w)
Supplement: Supplementary file 1 — Supplementary Information. [file 41598_2022_17785_MOESM1_ESM.pdf]

Supplementary Information

Hydrogel-extraction technique for non-invasive detection of blue fluorescent substances in plant leaves

Shigeyuki Iwasa[1,2,*], Yuso Kobara[3], Katsumi Maeda[2], and Kuniaki Nagamine[1,4]

[1] Research Center for Organic Electronics, Yamagata University, Yamagata 992-8510, Japan

[2] System Platform Research Laboratories, NEC Corporation, Chiba 270-1198, Japan

[3] Institute for Agro-Environmental Sciences, National Agriculture and Food Research Organization, Ibaraki 305-8604, Japan

[4] Department of Organic Materials Science, Yamagata University, Yamagata 992-8510, Japan

*email: s-iwasa@yz.yamagata-u.ac.jp

Supplementary Information

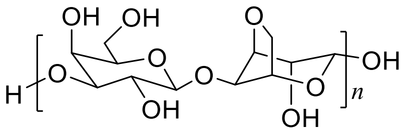

Figure S1. Chemical structure of an agarose polymer, which is the main component of agar.

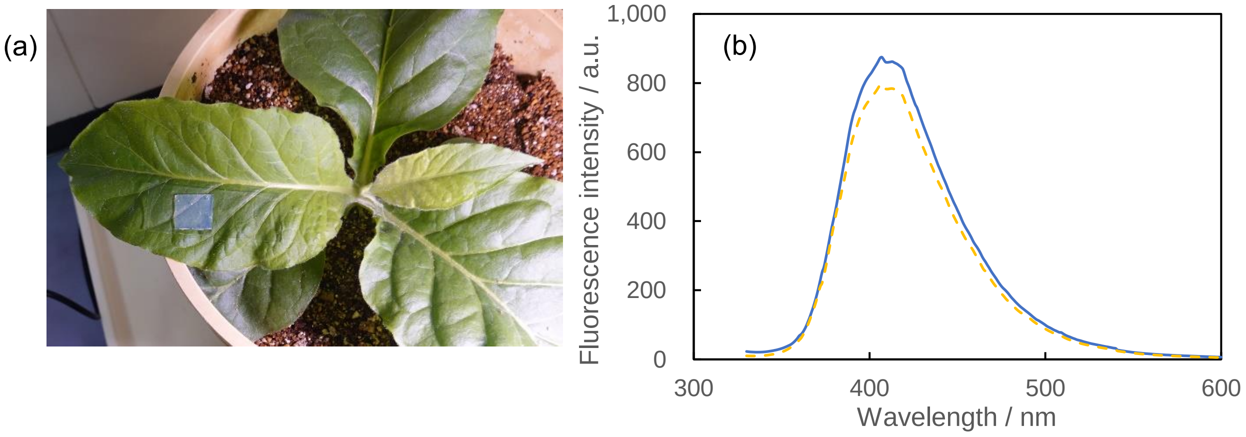

Figure S2. (a) Photograph of hydrogel extraction: a hydrogel film extracting salicylic acid from a tobacco leaf. (b) Fluorescence spectra of a hydrogel film after its placement on a tobacco leaf for 3 h (blue line). Three hundred mL of 7 mmol/L salicylic acid solution was poured over the soil 24 h before the extraction. Fluorescence spectra of a hydrogel film containing 0.2 mmol/L salicylic acid aqueous solution (yellow dashed line). Fluorescence emissions were obtained using UV light (Xenon lamp, 310 nm).

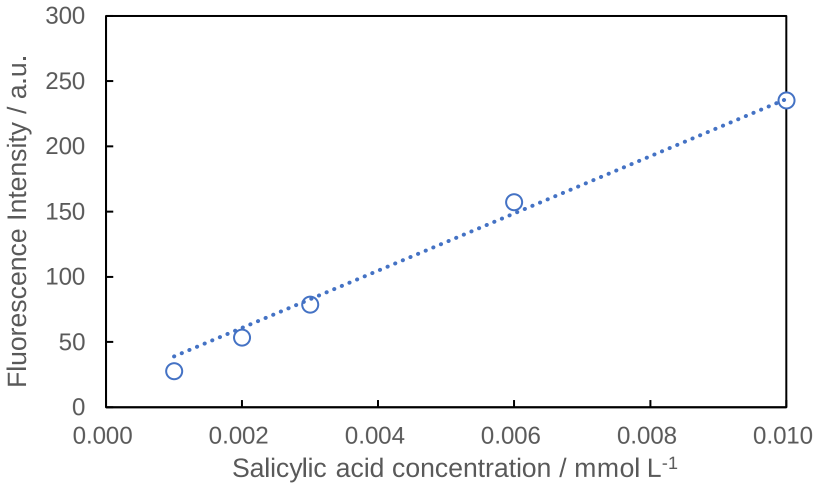

Figure S3. Calibration curve: fluorescence intensity at 410 nm depending on salicylic acid concentration in methanol solution. Fluorescence emissions were excited by UV light (Xenon lamp, 310 nm).

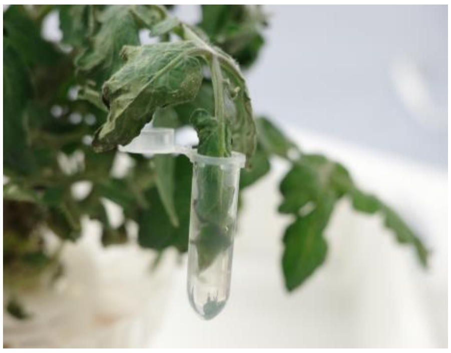

Figure S4. Photograph of the water immersion of a leaf in a plastic tube.

Supplementary Information

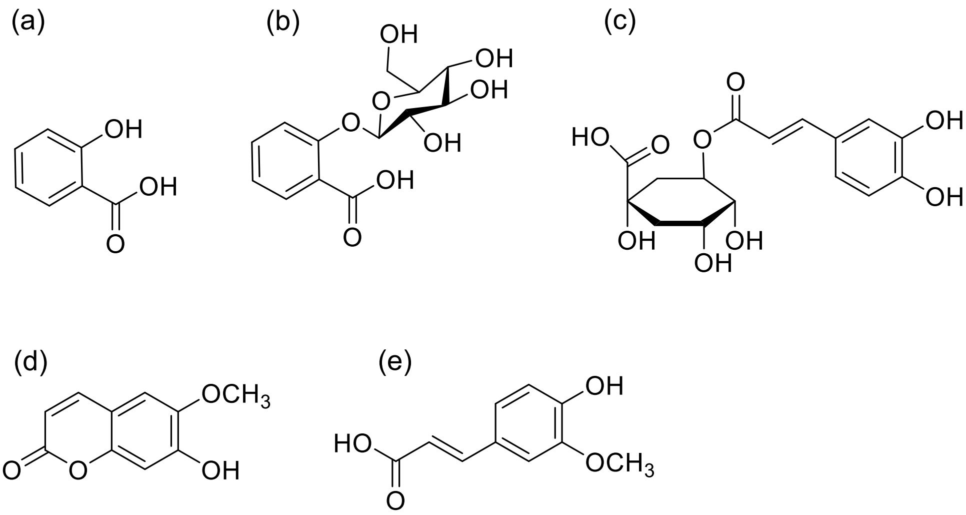

Figure S5. Chemical structures of (a) salicylic acid, (b) salicylic acid 2-O-β-D-glucoside (SAG), (c) chlorogenic acid, (d) scopoletin, and (e) ferulic acid.

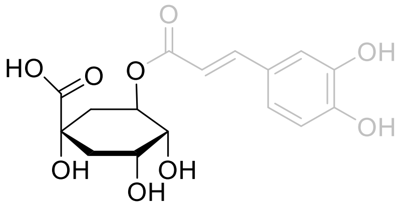

Figure S6. Fragment of chlorogenic acid.

Supplementary Information

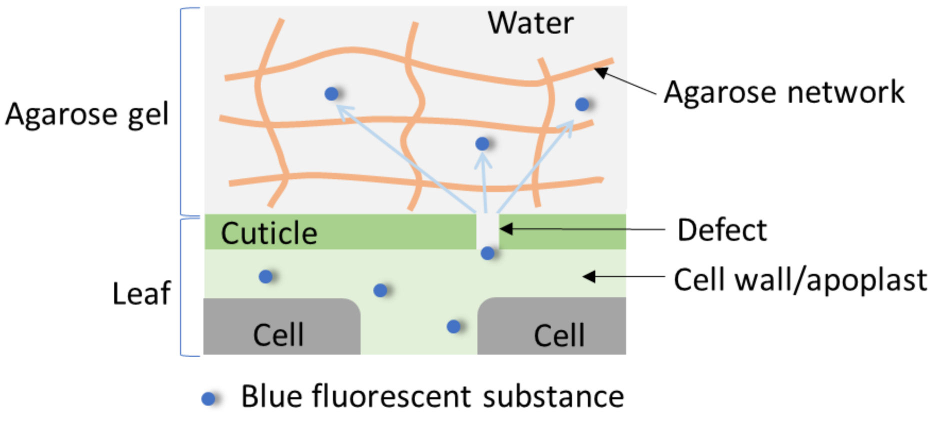

Figure S7. Conceptual diagram of hydrogel extraction from a leaf of infected cherry tomato.

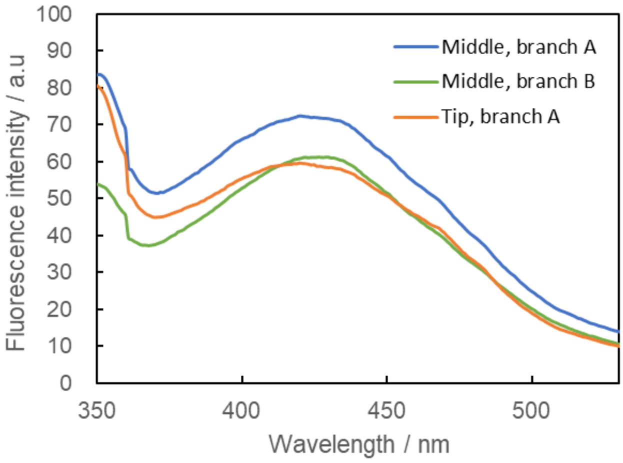

Figure S8. Fluorescent spectra of hydrogel films after the extraction of middle parts (blue and green) of leave of different branches (branch A and B), and the tip part (orange) of a leaf of branch A. Fluorescence emissions were obtained using UV light (Xenon lamp, 310 nm).
